# Authority and the Future of Consent in Population-Level Biomedical Research

**DOI:** 10.1093/phe/phz015

**Published:** 2019-10-30

**Authors:** Mark Sheehan, Rachel Thompson, Jon Fistein, Jim Davies, Michael Dunn, Michael Parker, Julian Savulescu, Kerrie Woods

**Affiliations:** 1 Ethox, Oxford University; 2 School of School of Sport & Exercise Science, Swansea University; 3 Inter-Disciplinary Ethics Applied Centre (IDEA), Leeds University; 4 Big Data Institute, Oxford University; 6 Wellcome Centre for Ethics and Humanities, Oxford University and Ethox, Oxford University; 7 Uehiro Centre, Oxford University; 8 Digital Division, Oxford University

## Abstract

Population-level biomedical research has become crucial to the health system’s ability to improve the health of the population. This form of research raises a number of well-documented ethical concerns, perhaps the most significant of which is the inability of the researcher to obtain fully informed specific consent from participants. Two proposed technical solutions to this problem of consent in large-scale biomedical research that have become increasingly popular are meta-consent and dynamic consent. We critically examine the ethical and practical credentials of these proposals and find them lacking. We suggest that the consent problem is not solved by adopting a technology driven approach grounded in a notion of ‘specific’ consent but by taking seriously the role of research governance in combination with broader conceptions of consent. In our view, these approaches misconstrue the rightful location of authority in the way in which population-level biomedical research activities are structured and organized. We conclude by showing how and why the authority for determining the nature and shape of choice making about participation ought not to lie with individual participants, but rather with the researchers and the research governance process, and that this necessarily leads to the endorsement of a fully articulated broad consent approach.

## Introduction

Large scale or population-level biomedical research (including big data, genomics and biobanking) has become one of the most important requirements for the long-term capacity of a health system that aims to improve the health of the population. There are significant potential benefits to population-level research, from economies of scale and increased statistical reliability to the enabling of studies that require scale to function. This includes seeking patterns to identify preclinical disease markers and establish penetrance of variants, and the ability to test hypotheses on large cohorts and to see small effects. The ability to conduct research at scale and depth has increased rapidly in the last few years, based on the availability of new technologies and methods that are increasingly sophisticated.

This form of research raises a number of well-documented ethical concerns which do and will continue to haunt the progress of this research in important ways. Perhaps the most significant of these concerns is the unfeasibility of the researcher obtaining fully informed specific consent from participants. In what follows, we focus on this ‘consent problem’, in light of the fact that the potential future benefits of this kind of research depend on its scale and efficiency in ways that run directly counter to the requirement of participant informed consent as currently understood.

Two proposed technical solutions to the problem of consent in large-scale biomedical research that have become increasingly popular are meta-consent (Ploug and Holm, 2015, 2016) and dynamic consent (Kaye *et al.*, 2015). We begin by critically examining the ethical and practical credentials of these proposals and find them lacking. We then revisit an older model which provides a simpler and more effective solution to facilitate research without compromising either the ethics or the potential benefits of future research. Our critique of meta-consent and dynamic consent as solutions focuses on the claim made by both to be new, ethically warranted ways of addressing the tension between facilitating effective large-scale research and obtaining individual specific consent. We suggest that the ‘consent problem’ is not solved by adopting a technology-driven approach grounded in a notion of ‘specific’ consent, which is unachievable and likely to increase the workload of participants and researchers, but by taking seriously the role of research governance in combination with broader conceptions of consent.

The broad consent model that we propose revisits aspects of the research governance and consent debate that have been missed in recent discussions. This model, in our view, has been all too hastily set aside, perhaps because it appears technically less sophisticated or because it is not quite so overtly driven by an aim of maximizing choice. We suggest, however, that the mistaken assumption underlying the dynamic and meta-consent models is that choice making around research participation should necessarily always reside with individual participants. In our view, this misconstrues the rightful location of authority in population-level biomedical research activities. We conclude by showing how and why the authority for determining the nature and shape of choice-making about participation in such research ought not to lie with individual participants, but rather with the researchers and the research governance process, and that this necessarily leads to the endorsement of a broad consent approach.

The relationship of consent to autonomy, and the nature of autonomy, are central to our arguments. The reason that we require consent, both historically and theoretically, is to preserve an agent’s right to autonomous choice (Faden and Beauchamp, 1986). Autonomy is self-determination, which involves forming and acting on a conception of the good life (or a life plan) and values, free from the interference of others (Young, 1982), and many choices we make can undermine our autonomy or fail to respect it appropriately (Savulescu, 1994; Savulescu and Momeyer, 1997). Our point will be that autonomy can be consistent with taking part in an open-ended project, just as it can be consistent with a precisely defined or constantly evolving consent process. Marriage involves a commitment to an uncertain future. So too can research participation. Autonomy is respected when a participant is given the opportunity to give or withhold broad consent, provided they understand the nature of broad consent and the gravity of what is at stake in participating in population-level research studies, broadly construed.

## Examples of Population-Level Research

The following examples are useful in understanding the kinds of research to which these issues apply, and in pinpointing the kinds of methodological issues that are relevant for obtaining valid consent to population-level research.

Longitudinal, data-intensive research-related projects run for extended periods of time in order to be able to model and predict longitudinal patterns of disease over many years. These projects may be relatively specific, for example, relating to a single disease area or may be more general, covering a range of medical conditions, social and economic factors etc. Such studies exemplify the kinds of potential issues relating to consent in population level research. In such studies, the researcher cannot predict all of the potential uses of the data in advance, given the evolving nature of medicine, data science and computational power. Over time, the researcher may need to keep track of participants who may be ‘lost to follow-up’ or who may have died, particularly if re-consent is required. Such studies are dependent for their scientific validity on consistent cohorts, maintained over the long term to ensure populations can be tracked over time so that meaningful comparisons and conclusions can be drawn from the data. This need for stability implies a certain type of relationship with the participants, where the broad expectations are explained at the outset, with the understanding that participants will generally continue to allow use of data about them for the duration of the study. For all these reasons, when an opportunity arises for a potential new data use or research activity, the researcher must decide whether a particular sub-project (or research activity) is in line with the original consent. Examples of research of this type include UK Biobank, The Cooperative Health Research in South Tyrol (CHRIS) study and Avon Longitudinal Study of Parents and Children (ALSPAC) outlined briefly below.

UK Biobank is a resource for population scale longitudinal studies based on an initial recruitment aim of 500,000 people aged 40–69 at recruitment. UK Biobank collects lifestyle and environmental information, medical history, physical measurements, and biological samples from participants over the life of their involvement in the study (UKBiobank, 2007) CHRIS study is a longitudinal population- based study that began in 2011. It investigates the genetic basis of common chronic conditions associated with human ageing. It also looks at interactions between genetics, life-style, and environment in the South Tyrolean population (Pattaro *et al.*, 2015).

The Avon Longitudinal Study of Parents and Children (ALSPAC), or ‘Children of the 90’s is an ongoing longitudinal study of children of mothers who were pregnant in the early 1990’s in South West England, and ‘the overall objectives of the study are to understand the ways in which the physical and social environments interact over time with genetic inheritance to affect health, behavior and development in infancy, childhood and then into adulthood’ (Golding & ALSPAC Study Team, 2004).

## Dynamic Consent and Meta-Consent

Informed by the illustrative examples of population research above, we begin by reviewing the advantages and disadvantages of dynamic and meta-consent as solutions to consent in such research.

Dynamic consent is an IT-based solution developed in response to challenges presented by consent to biobank research, with potential application in other settings that its proponents argue require ‘re-consent’. It is dynamic because it involves multiple interactions, allowing participants to consent to new uses, or to change their existing permissions (Kaye *et al.*, 2015). Participants may choose to opt in or opt out of being re-contacted and opt in or out of new research. They might, for example, (i) give a broad consent to participate in all research activities, (ii) give broad consent for some research activities but not others and then to have increasing engagement either about re-contact or about consent to future research if they wish; or (iii) to pause their interactions with the research, potentially resuming at a later date. Dynamic consent is the idea that control for all choices and all engagements can be given to, and left up to, participants.

The implicit rationale for the adoption of dynamic consent is that it is ethically preferable because it allows participants more choice and more control (and so perhaps more exercise of autonomy). Dynamic consent aims to enable participants to tailor their involvement as opposed to ‘all or nothing’ or ‘one-off’ consent or withdrawal (Kaye *et al.*, 2015; Williams *et al.*, 2015).

Meta-consent, by contrast, was developed in response to questions about whether it is always necessary to seek informed consent for secondary health-related research (Ploug and Holm, 2015, 2016). Meta-consent enables individual decisions about how to approach consent to future secondary use of both existing and future data. It functions by allowing individuals to express their personal preferences regarding the type(s) and frequency of consent decisions—giving them putative control over precisely how consent will continue to be sought from them on an individualized basis. The meta-consent proposal is designed to be instigated as young adults gain full legal rights with detailed information about each potential consent type, and the implications of choosing each type, to be provided to them at the point of seeking an initial meta-consent. Subsequently, the meta-consent proposal requires individuals to be enabled to continually review and potentially revise their expressed consent procedure preferences throughout the life course, with the use of reminders to update consent preferences every few years.

Because it allows individuals to control the kind of consent that they give, meta-consent claims to better respect participant preference (e.g. preferring not to give further consent) and therefore better respect autonomy, than dynamic consent, but the choice is still from a range of predetermined options. Meta-consent aims to improve consistency, information and deliberation about consent decisions (Ploug and Holm, 2016).

## Criticisms of Dynamic Consent and Meta-Consent

These two new approaches to consent for population-level biomedical research have received some attention, both positive and negative. In what follows below we outline the concerns that have been raised and critically appraise them.

We concentrate on criticisms of dynamic consent because we understand meta-consent to be a species of dynamic consent. Ploug and Holm allow multiple (presumably unlimited) changes to the specific details of the consent given by an individual. This means that even if the scope of the choices is limited to the form that future consent will take, that choice is dynamic and can change as often or as little as the participant wishes. Thus, while being rightfully understood as a version of dynamic consent, meta-consent differs in the sense that it focuses more specifically on allowing the participant to dynamically control the kind of consent that they favor.

Notably, both dynamic consent and meta-consent approaches seem to allow some control of the process to reside with the researcher to some extent. This is clearest in the meta-consent case, which seemingly permits researchers to offer a limited range of consent models to prospective participants. The dynamic consent approach seems, at various points, to allow the dynamism to be curtailed and restricted in various ways, thus taking choice and control away from the participant. In both cases, the approaches are silent about why this is the case and precisely when the control accorded to participants should be limited in this way. We return to this below in authority section, in the context of what we call the Authority Problem.

Dynamic consent has been criticized in various ways. We have grouped these criticisms into four headings which we discuss in turn.

### Practicalities

Dynamic consent claims to be an easy to use and accessible system that facilitates research through greater participant engagement. In the current technological context, the value of being able to deliver this kind of participant engagement platform is clear. It is also clear that some research in the future will require an active and ongoing supply of data from participants and so a dynamic consent approach will require a highly interactive technological platform.

The main practical questions surround the ability to conduct research in an efficient and manageable way, while remaining true to the task of obtaining a full range of choices about consent and, where required, fully informed specific consent.

In order for participants to be able to express their preferences fully, the advocates of dynamic consent argue that participants need to be aware of the potential choices. This presents two potential problems. First, the researcher must decide whether a particular use is captured by the original consent or not. This judgment can require interpretation, even if the initial consent was quite specific. Second, the participant must decide how they might wish to be informed about any potential uses and how they might wish to express their preferences. If a ‘push’ mechanism is used, participants may be bombarded with an excess of potential choices, may experience ‘consent fatigue’ and so may disengage from the process. If a ‘pull’ mechanism is used, expectations would need to be clear about the rates of change/addition of uses so that participants have an appropriate time to respond, before the new use is started.

Aside from the decision of when to offer participants new choices, there are a set of issues (and more choices for participants) about how choices should be expressed and the information provided. Healthcare research activities are complex and it may be difficult or impossible to adequately explain the nature, purposes, and consequences of particular choices to inform the participants in ways that are meaningful to them. A huge volume of choice and information might well lead to ‘consent fatigue’ with people either opting out completely, just opting in without reading the options, or simply not responding. To be clear, this may not prove to be a problem for researchers or participants in practice, but may give rise to a kind of broad or blanket consent by default, which the model was designed to avoid.

There may also be some related technical barriers associated with making and enacting choices. The assumption in dynamic consent models is that choices can be made in (near) real-time using technological platforms to record and propagate preference choices. Such mechanisms, and particularly those concerned with the meta-consent proposal are likely to be both costly and burdensome (Manson, 2019), and may introduce biases as they depend on a certain level of technical competence which may exclude participants without computer access or in particular demographic groups. There may be a time-lag between the making of the choice and its enactment which is at odds with participants’ expectations of how their choices should be honored.

What is most important about these practically oriented concerns is that they point toward the need for fine-grained ethical judgments that are importantly related to the kinds of consent that we take to be ethically required. There are clearly ways around many of these problems, but they turn on making decisions about just how many decisions and how much detailed, interactive information is taken to be important for participant choice. We can always provide more choice and more interactivity: the question is where we take the ethical requirement for information provision and specific choice to run out.

### Capturing Preferences and Obtaining Specific Consent Properly

Ploug and Holm (2015) point out that some participants would in fact prefer not to make some choices (about some kinds of research) and that allowing this preference to be satisfied would overall allow more preferences to be satisfied in the way in which participants engage with the research (Wallace *et al.*, 2015).

This set of criticisms claims that dynamic consent appears to force participants to engage with the research and make choices in ways that go against their preferences. As we saw above, this seemed to be a key practical issue. Steinsbekk, Myskja, and Solberg (2013) and Ploug and Holm (2015) argue that repeatedly asking for consent might in fact reduce engagement by routinizing the consent process. Requiring participants to constantly revisit consent does not respect the autonomy of the person who just wants to support research and have no further contact. Moreover, it may turn out that the vast array of choice, and the constant barrage of information, could undermine the very choices being made: allowing people to choose more may make those choices less informed and or lesser quality (Human Research Authority & Human Tissue Authority, 2018).

However, as we have seen, dynamic consent does not require participants to re-consent every time: they can opt in or opt out of making a choice. Indeed, the dynamic consent model explicitly includes provision for participants to make choices about their level of interest in consenting (meta-consent) (Kaye *et al.*, 2015; Williams *et al.*, 2015).

While there may well be persistent questions about the ability of dynamic consent to meet the standards required of fully informed specific consent, proponents of dynamic consent have a clear response to this set of criticisms, in one respect at least. The dynamic consent model (and what makes meta-consent a version of dynamic consent) allows for dynamism of precisely the sort that it is being criticized for omitting. Dynamic consent is not to be understood as simply the view that individuals are permitted (or required) to make choices about all research which intends to make use of their data. Instead, it very clearly allows for multiple levels of choice on the part of the participant. The participant can switch on or switch off as and when they feel inclined to do so: they can alter their specific choices, they can alter their choice about the kinds of choice that they wish to make, and they can adjust the kind and level of detailed information that they are provided with in regards to each research use (Kaye *et al.*, 2015).

### The Ethical Justification of Dynamic Consent and the Inferiority of Broad Consent

Beyond the supposed value of enabling a more fine-grained set of choices, the supposed ethical impetus behind dynamic consent is that more choice or more control is more ethical (and importantly, that less choice, fewer choices and/or less control is unethical or ethically inferior). This position is tied to debates about the adequacy of broad consent models of consent to population-level research programmes. If it were true that broad consent failed to respect the autonomy of participants or correspondingly, and that specific consent was the only kind of consent that was ethically acceptable, then dynamic consent would represent a genuine, ethical solution to the problem of consent to population-level research.

However, the claim that broad consent, without the option of more specific consent if desired, is unethical or ethically inferior is unwarranted. It rests on the mistaken idea that only specific consent can count as valid (fully informed) consent—the fallacy of specific consent (Sheehan, 2011). Importantly, the presumption that more choice is more ethical overlooks the fact that we make perfectly acceptable broad choices on a routine basis without our autonomy or freedom being undermined in an unethical way. We routinely make decisions that restrict, limit or change the choices that we make in the future. And, importantly, we routinely decide to allow others to make decisions that affect us in important ways. Broad consent to allow others to make decisions on our behalf or according to a settled process are commonplace and ethically unproblematic.

The argument, then, for the ethical acceptability of broad consent rests on the simple observation that we routinely and unproblematically make decisions that limit or delegate future decisions. Asking a friend to order for us at a restaurant is just one example (Sheehan, 2011) of a choice that we can delegate. Once we see that we commonly make these kinds of choices and that they are ethically acceptable, we can also see that the information that we require for those choices is radically different from the information required for a specific choice. If we are delegating a choice, we (should) want to know, for example, how the chooser will make a choice on our behalves in the future, what kinds of general values will be relevant to that choice, and what the goals are that the choice will aim to achieve.

The idea here is that we see a particular kind of focus on specific choices to the exclusion of other kinds of choices. As Manson (2019) has compellingly argued, we are trapped in a simple clinical model of choice making that limits how we think about informed consent in our bioethical treatment of population-level and biobanking research. The claim here is that we presume that respect for autonomy in this context requires an unfettered requirement to also *promote* participants’ autonomy. However, giving participants full control over decision-making in this way mis-conceptualizes the underpinning connection between the value of autonomy and procedural requirements for obtaining valid consent from participants. Thus, because broad consent is just as ethically acceptable as specific consent, dynamic consent lacks an ethical imperative for giving participants more choice; proponents of the model commit the fallacy of specific consent by equating expanded choice and re-consent with a requirement to promote autonomy without justification.

However, proponents of dynamic and meta-consent have something of a response here. After all, both models allow for broad consent to be given by participants and it might turn out that the vast majority of participants opt for broad consent rather than the constant interaction associated with many specific consents. This is because dynamic consent is better thought of as an enrollment approach within which specific and broad consents are permissible.^1^ The question then remains to justify dynamic consent as an enrollment approach. We return to this question in the next section.

### Resources, Research Benefits and Other Values

Finally, both dynamic consent and meta-consent fail to pay sufficient attention to the fact that respect for autonomy is only one value amongst others. Even if giving more and more choice did indeed promote autonomy, whether autonomy ought to be promoted in this way is an ethical issue that involves weighing this value against effective use of limited resources and the moral imperative to conduct research (Thorogood and Zawati, 2015). To give a trivial example, some participants might like to choose the sex or dress of the doctor taking their blood, but this is not a choice that they ought to be given in the context of other (perhaps, any) constraints.

Participants need to give valid consent to the procedures they are undertaking but it is a further question whether there is an ethical requirement to obtain specific consent for future uses of data or biological materials. The creation and maintenance of bioresources and databanks and the infrastructure that supports them is hugely expensive. In many cases these are created with public money. The creation and sustainability of such resources is in the public interest. Here, the tradeoff may well be that there are certain kinds of uses of data, for the public interest, that outweigh the requirement to obtain any kind of consent. This is particularly true if we attend to the structures and governance of the research institutions that will be permitted to conduct research using this data. As we suggest below, governance is important in these settings and can help to manage risks and confidence in research and research institutions without the need for choice-intensive models of participation.

## Authority

Thus far, we have considered a number of objections to both dynamic and meta-consent. These objections are illuminating but they do not fully settle the issue. Very briefly, it looks to us as though the most plausible understanding of the meta-consent approach is that it is a more closely specified version of the dynamic consent approach. Second, although there are definite practical concerns about the dynamic consent approach, these can be dealt with—perhaps with some costs to efficiency and financial/opportunity costs. People will disagree about how significant these costs are in relation to the purported benefits. Third, there are some more general worries about the quality of the individual participant’s consent in the face of a proliferation of choice. But it may be that the participants themselves are able to monitor this and adjust their involvement accordingly. Fourth, the impetus toward dynamic consent (and away from broad consent) seems to rest on the idea that broad consent is ethically inferior to specific consent. We have argued that this is a mistake, but also recognize that dynamic consent models can include broad consents and so the degree of disagreement here might be limited. Finally, we pointed out that respect for autonomy is one among many values that are at stake in population-level biomedical research, and that consequently there may be contexts where the value of the research takes precedence over the requirement to facilitate specific consent in every case.

In this section we present a distinct and, we suggest, more pressing objection to dynamic (and meta-consent) which goes to the heart of broader uncritical trends towards maximizing participation and choice for its own sake. This objection turns on a question of authority: how can we justify giving authority for determining the nature and shape of choice about participation to individual participants (in addition to the choice to participate)? This question is not explicitly considered in the literature and, once we reflect on it, we see that it is difficult to answer and has serious implications for research. We go on to argue that this authority, as it is rightfully understood, should rest with the researcher and the research governance process, thus allowing the participant to make an initial decision to participate and, as usual, to hard-withdraw at such time as they no longer wish to be involved.

Recall that in the section above we argued that broad consent is not ethically inferior to specific consent, and so there is no general ethical reason to prefer specific consent over broad consent. These two models of consent are different and require different sets of information in order to be properly informed. Importantly, we have also seen that both dynamic and meta-consent include broad consent as possible options for participants entering research. So not only is there a good argument for thinking that broad consent and specific consent are ethically equivalent, the proponents of dynamic and meta-consent have built both options into their systems, thus agreeing with their ethical equivalence (via permissibility: why would we allow participants to choose an ethically inferior form of consent?).

But the proponents of dynamic consent clearly still believe that their approach is preferable to the simpler, broad consent model. This must be because they take there to be added (ethical) value attached to giving participants control (and hence authority) over the nature and shape of their interaction with the research rather than allowing the researcher (and the governance process) the authority to determine the nature and shape of that interaction, which is then chosen (or not) by the participant.

This is the authority problem: why think that the participants should be in control of the nature and shape of their interaction with the research?

Both the dynamic and meta-consent approaches largely accord control over the nature and shape of the choices that are being made by potential research participants’ choices to individual participants. It is not clear that this move is ethically justified. Participants should retain the right to choose whether they take part in research or not, and have a right to withdraw if they do take part, but it does not follow from this that they have the authority to construct the design of that research or their engagement with it. Locating authority entirely with participants overlooks the role of expertise, particularly of the researcher, in shaping the research. The following three examples help us to see the problem and the need for justification here.

Consider first consent to airline travel: As airline passengers we consent to behave in particular ways with the expectation that we will be kept safe and arrive at our chosen destination. Once the plane has begun to taxi, we may not ordinarily withdraw our consent to flying or decide that we would like the plane to go somewhere else. We have committed to a process and will not be asked for consent to changes that may occur past a certain point. We are only given the authority to choose whether to accept the prescribed conditions or not—these conditions are presumably about safety and efficiency. In this case, ‘participants’ are not given the opportunity to choose dynamically; consent to fly to a particular destination is a process that necessarily ends at the point the plane begins to taxi.

A second example in this vein involves consenting to voluntary service overseas (VSO). Here we would expect that a volunteer may not ordinarily withdraw their consent to participate past a certain point of engagement. They have committed to a programme of work and there are implications not only for themselves, but for other participants and potential beneficiaries, if they were to withdraw. All volunteers are given appropriate information and time for consideration prior to joining up, and, once past a certain point, are discouraged from reneging on their commitment or deciding that they would rather be located in a different country or undertaking a different kind of aid work. Here too individuals are free to choose to commit to the programme but they are not given control of the nature of the programme itself or the permission to withdraw at any time for any reason. Importantly in this case, the authority to shape the volunteer programme and what it involved lies with the programme organizers. The ability of the programme to deliver aid and support of various kinds depends on the reliable commitment of the volunteers to the programme (as it is designed by the organizers).

Both of these examples are clear cases where the authority for choice construction is not given to individual consenters even though the choice to participate still firmly remains with them. In the air travel case, passengers are simply not given the choice: that is, they have no authority to decide about the flight, where it goes or their involvement in it, once the plane has left the gate. In the VSO case, the rationale for requiring an upfront decision rather than allowing dynamic choice lies with the nature of the enterprise and the authority to determine the programme. The success of the VSO programme depends on the ability of the organizers to count on the volunteers not to renege on the choices they have made.

Of course the VSO case is subtle in important ways. Under certain special circumstance it might be perfectly appropriate for a volunteer to discuss possible changes to their participation in the programme or to withdraw. It might, for example, turn out that the volunteer feels uncomfortable being the only foreign woman in a predominantly male village. This subtlety underlines the central point about authority: sometimes changes to the nature of the participation can be justifiably negotiated, but this is in the context of well-defined authority.

We can contrast these two cases with a third: the pick-and-mix sandwich shop. There is clearly a significant advantage to the model of the sandwich shop which allows customers to choose any variety of sandwich toppings (cheese, lettuce, salami etc.) and ‘sandwich forms’ (sliced bread, bun, warmed, with accompaniments, etc…). When it comes to our sandwich lunch, more choice and more control over the range of choice looks better. We rightly think that patrons of a sandwich shop are the authority when it comes to their own lunchtime eating preferences and so it makes sense to offer this kind of range of choice. For those who are not so concerned about too many specific choices, the sandwich shop may well offer a range of pre-set sandwich options too—where some authority for sandwich construction remains with the shopkeepers.

In each of these cases it is clear why the authority to construct the range of choice lies where it does: the sandwich shop succeeds or fails on the back of customer choice, In contrast, the safety and efficiency of airline travel requires a fixed choice, and the ability of the VSO programme to deliver aid and support of various kinds depends on the reliable commitment of volunteers. In the case of population level research, it is unclear why a dynamic consent model should be preferred. Alternatively, it is unclear why population level research should be thought of as something like consumer-led activity.

The key to our criticism of dynamic consent (and meta-consent) is the unexplained and unjustified assumption that the authority to construct and shape the research, the participants’ involvement in it, and the authority to choose to participate, lies with the potential participant rather than the researcher and the research institution. In order for research to be conducted in an ethical, efficient and most effective way the authority for shaping the research and for constructing the choice given to potential participants should lie with the researcher and an appropriately expert research governance process. This does not rule out more choice-intensive consent processes but it is clear about where the locus of the authority to determine these processes should lie. Importantly, potential participants are still given a fully informed choice which respects their autonomy: they are presented with a research, governance and enrollment arrangement and choose to be a part of it or not. The issues here share important similarities with the VSO example: research can be jeopardized, and made less efficient or less effective, if participants alter their preferences mid-stream. Just as the vulnerable people who stand to be helped by the VSO programme would suffer if volunteers’ preferences are accommodated without consideration, so future patients would suffer from poorly conducted research. There is a right to withdraw, but this right can be structured in advance of a decision to participate, and importantly, the authority for this structuring does not lie with the participant.

The research enterprise is not an essentially competitive, ‘consumer choice’ situation. It is a social endeavor aiming at systematically improving the health of future patients. It is a goal driven activity that aims at the production of valuable knowledge. It is methodologically complex and requires significant knowledge and training in order to make incremental advances in any given field of inquiry. Because of these key features, the authority for the design of the offer is intimately dependent on the aims of science and lies with the researchers and the governance of research. Patients and their interests are far from irrelevant in this enterprise, but they are entering a realm of activity in which they are not the authority. We propose a ‘new’ approach to the question of consent to large scale or population-level biomedical research: a combination of broad consent to governance with participants retaining the right to withdraw from future research.

## Consent for the Future

**Figure 1.**
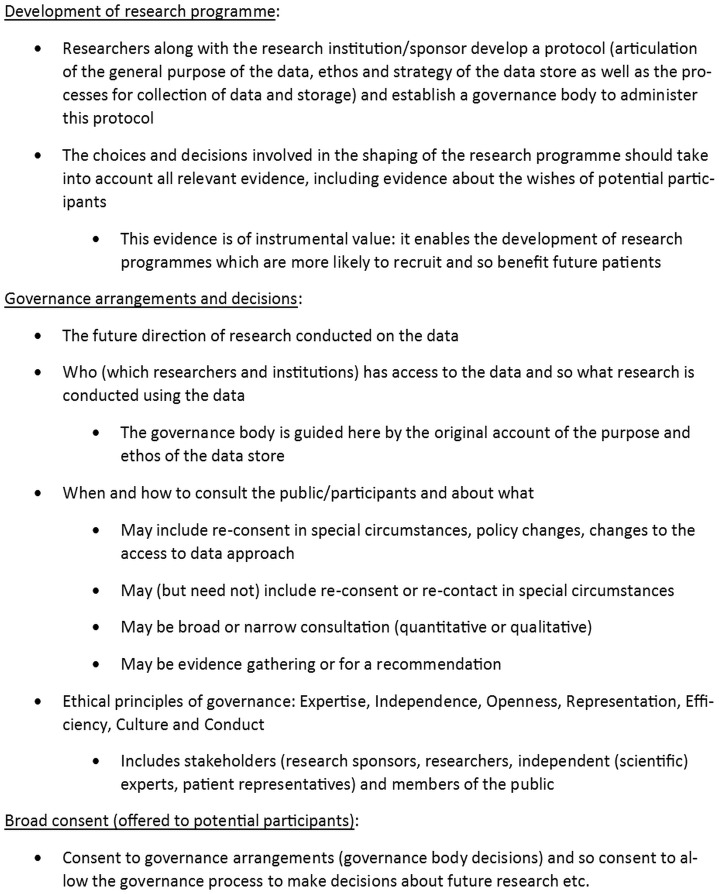
Consent for the future: general principles.

We have suggested that the future of consent in population-level biomedical research requires exploring the limits of the authority of the participants’ role in shaping and controlling the research. We argue that that current trends move in a mistaken and unjustified direction: researchers and research governance structures are by their nature vested with an authority, and should draw on expertise to determine the nature and progression of research. We need to rethink the future of consent to research at the population-level and create systems of reflexive governance (See Figure 1).

There are general principles for the future of consent that need to be recognized here. In developing the research programme, researchers, along with the research institution or sponsor, should produce a protocol (which will include an articulation of the general purpose of how the data will be used and stored), and they ought to establish a governance body to administer this protocol. The choices and decisions involved in the shaping of the research programme should take into account all relevant evidence, including evidence about the wishes of potential participants. This evidence is of instrumental value: it enables the development of research programmes which are more likely to recruit participants and so maximize the chance of benefiting future patients. Consultation may be broad or narrow (quantitative or qualitative) and may be evidence gathering, or focused on developing a recommendation for how the governance arrangements should operate.

Governance arrangements and decisions will incorporate matters concerning the future direction of research conducted on the data, as well as who has access and therefore what research is conducted using the data. Here, the governance body is to be guided by the original account of the purpose and ethos of the data stored. Decisions about when and how to consult participants and the public may include re-consent in special circumstances. These would include policy changes and changes to data access approach. Ethical principles of governance are/should be: expertise, independence, openness, representation, efficiency, culture and conduct (Sheehan, Marti, and Roberts, 2014) and include all stakeholders (research sponsors, researchers, independent (scientific) experts, patient representatives) and members of the public.

Potential participants should be offered broad consent. Participants will decide whether to consent to governance arrangements (governance body decisions) and so consent to allow the governance process to make decisions about future research etc. Participants retain the ability to withdraw according to the terms of this broad consent. Depending on the context, this process of withdrawl may include complete, hard withdrawal, or simply withdrawal from future uses. Communicating information about what is happening with the research as it evolves is integral to a reflexive governance model. The information that ought to be provided includes updates, activities and studies available for participation, but is not about choice.

This future approach to consent is not one where participants do not have any choice, but it is one which is embedded within the context of the socially governed, researcher-led institutions that have appropriate expertise and authority to construct and conduct research that produces public benefit. In considering the potential harms associated with large-scale biomedical research, we should include harm caused by not making further use of the data collected. A large proportion of medical data are not used or shared effectively (Jones *et al.*, 2017). There is new potential for harms appearing with increasing complexity (associated with a shrinking of number of participants who can properly understand the processes) and this is reflected in the model we propose. There is a need for research governance that has public engagement embedded where necessary, but that allows those with expertise to make decisions regarding research on behalf of participants. Fundamentally, participants voluntarily agree to be a part of an institution that makes decisions about the future as it becomes the present. This is not a specific consent to future research but a perfectly reasonable consent to fair and transparent methods of governance and decision-making.

## Objection: Unpredictable Future

The future is highly unpredictable, and this has particular implications for the uncertain and ongoing evolution of population-based research that have taken place over recent years, and that will continue to take place into the future. For example, the value of whole genome sequencing of all samples in research biobanks was not anticipated. Nor was the rise of public-private partnerships that has increasingly seen big pharma investing in biobanking research funded originally through public investment. The radical increase in data sharing in population-based research was also unexpected, as has been the emergence of whole exome sequencing that raises new questions about how results that offer potential benefits to participants should be returned (Lucassen, Montgomery, and Parker, 2017).

On one interpretation, broad consent might seem to struggle with rapid and important changes of these kinds. If broad consent is understood as a paper-based consent that provides no easy way of re-contacting participants, obtaining re-consent, or providing further information and updates (without huge costs each time), then this concern would be well-founded. Equally, and in contrast, implementing a dynamic consent approach would make it far more straightforward to researchers to inform and interact with their participants. It might be thought therefore that the level of interest in dynamic consent is based in the fact that it offers an efficient way of addressing practical concerns that arise in a fast-moving area of research while ensuring that the benefits of research are realized to the greatest extent.^2^

However, such an interpretation of broad consent should be resisted. Broad consent can be as flexible and accommodating of technological evolution as the dynamic consent model. Under a broad consent model, researchers can introduce any re-contact, re-consent or update arrangement they see fit to serve the purposes of research. The main difference is that the design of this dynamic interpretation of broad consent is researcher-driven not participant-driven. Dynamic consent risks becoming unmanageable in practical terms; under broad consent, researchers can design flexibility that is both appropriate to the goals of the research and its public value, and benefit participants as appropriate. For example, many research protocols now feedback incidental findings, and there are certainly arguments in favor of this approach (e.g. Schaefer and Savulescu, 2018). However, there is also nothing wrong about refusing to feedback incidental findings provided this is made clear at the time of consent.

As we have argued, dynamic consent does not necessarily maximize autonomy. But even if it did, researchers are not under an obligation to maximize participants' autonomy or well-being at all costs—there are other values at play.

Will this be sufficient for public trust and transparency? That of course is a challenge. But, given the vast benefits research has produced for the public good and the general ethical nature of most researchers, researchers should be invested with the power to show their colors.

## The Future of Consent and Governance

We have suggested that the future of consent in population-level biomedical research requires exploring the limits of the authority of the participants’ role in shaping and controlling the research. We argue that that current trends move in a mistaken and unjustified direction: researchers and research governance structures are by their nature vested with an authority and should use expertise to determine the nature and progression of research. In so doing, consent to research at the population-level needs the creation of systems of reflexive governance (Porsdam Mann *et al.*, 2016).

This future is one where the autonomy of participants is respected by giving them a choice to participate in open-ended research, where the limits are defined in a broad consent. Such research is designed by researchers and institutions which are responsible to society. This involves the use of appropriate expertise and authority to construct and conduct research that produces public benefit. There are many settings where there is no opportunity to repeatedly change one’s mind once committed to a process, and we have argued here that population level research is, for the most part, one of these settings.

## Footnotes

1. “Dynamic consent is more than just another type of consent. It is a communication and engagement approach that could accommodate any of these consents depending upon the wishes of individual participants and the particular circumstances of the research project.” (Budin-Ljosne, 2015; Budin-Ljosne *et al.*, 2017).

2. We thank one anonymous reviewer for this objection.
